# Let It Grow: The Role of Growth Factors in Managing Chemotherapy-Induced Cytopenia

**DOI:** 10.3390/curroncol31120596

**Published:** 2024-12-21

**Authors:** Ruah Alyamany, Ahmed Alnughmush, Hazzaa Alzahrani, Mansour Alfayez

**Affiliations:** Department of Hematology, Stem Cell Transplant and Cellular Therapy, Oncology Centre, King Faisal Specialist Hospital and Research Centre, Riyadh 11211, Saudi Arabia or r.alyamany@hotmail.com (R.A.); aalnughmush@kfshrc.edu.sa (A.A.); halzahrani@kfshrc.edu.sa (H.A.)

**Keywords:** chemotherapy-induced cytopenia, growth factors, cytopenia, adverse effects

## Abstract

Chemotherapy-induced cytopenia (CIC) is characterized by neutropenia, anemia, and thrombocytopenia, which are common and serious complications in cancer treatment. These conditions affect approximately 60% of patients undergoing chemotherapy and can significantly impact quality of life, treatment continuity, and overall survival. The use of growth factors, including granulocyte colony-stimulating factors (GCSFs), erythropoietin-stimulating agents (ESAs), and thrombopoietin receptor agonists (TPO-RAs), has emerged as a promising strategy for managing CIC. However, the use of these growth factors must be approached with caution. This review provides an overview of the mechanisms, efficacy, and safety of growth factors in the management of CIC. Additionally, we discuss predictive markers for treatment response, potential risks, and highlight areas for future research.

## 1. Introduction

Cytopenia, or myelotoxicity, is among the most frequent off-target side effects of chemotherapy in solid malignancies and lymphomas not involving the bone marrow, typically manifesting as neutropenia, anemia, and thrombocytopenia [1]. Even with targeted and biological therapies, cytopenia continues to be a frequent complication.

The consequences are significant; neutropenia leads to recurrent infections and poor wound healing, anemia causes fatigue and reduced quality of life, while thrombocytopenia results in bleeding [2]. These complications often necessitate interruptions in chemotherapy or reduced doses, affecting disease response and overall survival [3,4]. Cytopenia may also prevent patients from undergoing critical, life-saving procedures [3,5,6].

Various strategies have been explored to address chemotherapy-induced cytopenia (CIC), with the use of growth factors showing promise.

Hematopoietic growth factors stimulate progenitor cells in the bone marrow to proliferate, mimicking the actions of the body’s natural erythropoietin (EPO), thrombopoietin (TPO), or granulocyte-stimulating hormones [7]. Growth factors may provide durable relief from chemotherapy’s toxic effects by promoting physiological recovery compared to transfusions or other temporary interventions. This review will provide a comprehensive overview of the available growth factors for managing CIC, with a primary focus on solid malignancies and lymphomas. Cytopenia in the context of myeloid malignancies and stem cell transplantation are not included, as they are typically a targeted effect in these settings, and the use of growth factors remains controversial.

## 2. Article Selection

In this review, a search was conducted through PubMed and ClinicalTrials.gov for publications and ongoing trials relevant to growth factor use in chemotherapy-induced cytopenia. The search was guided by keywords such as ‘chemotherapy-induced cytopenia’, ‘anemia’, ‘thrombocytopenia’, ‘neutropenia’, ‘growth factors’, ‘colony-stimulating factors’, ‘erythropoietin-stimulating agents’, and ‘thrombopoietin receptor agonists’. We included clinical trials, observational studies, preclinical research, and review articles from peer-reviewed journals, as well as registered clinical trials. Studies that examined the mechanisms, efficacy, and safety of growth factor use in chemotherapy-induced cytopenia, as well as the pathophysiology and risks of chemotherapy-induced cytopenia, were chosen. Only studies published in English were included. We excluded studies addressing cytopenia in other therapeutic contexts, such as radiation therapy and immunotherapy (e.g., CAR-T cell therapy). The search focused on recent studies and guidelines to ensure up-to-date insights, supplemented by earlier research for historical context.

## 3. Pathophysiology and Risk Factors of Chemotherapy-Induced Cytopenia (CIC)

The pathophysiology of CIC involves hematopoietic stem cells, which act as progenitors for granulocytes, macrophages, and erythrocytes. These stem cells have the unique ability to self-renew [1,8]. Under normal conditions, most of these cells remain inactive, i.e., they are not actively dividing. However, in response to the toxic effects of chemotherapy on circulating blood cells, more stem cells are forced into the active cell cycle to produce progenitors and replace the depleted blood cells [1,8]. Due to their rapid division, activated progenitor cells are particularly vulnerable to chemotherapy-induced destruction, unlike fully differentiated cells, which are more resistant [1,9,10]. The severity of CIC largely depends on the sensitivity and lifespan of the affected blood cells [1].

Although bone marrow typically compensates for the destroyed cells and recovers from cytopenia, some patients are more susceptible to prolonged cytopenia post-chemotherapy. Risk factors for prolonged cytopenia can be classified as disease-related, patient-related, and treatment-related (Table 1) [1].

## 4. CIC and the Use of Growth Factors (GF)

CIC is managed using synthetic growth factors (GFs), such as granulocyte colony-stimulating factors (GCSFs), erythropoietin-stimulating agents (ESAs), and thrombopoietin receptor agonists (TPO-RAs). Each type of cytopenia—neutropenia, anemia, and thrombocytopenia—requires a distinct management approach, as the effects of these conditions vary. CIC not only affects patients’ quality of life and performance status but also places a significant economic burden on the healthcare systems. Hospitalizations, transfusions, delays, and scheduling adjustments for chemotherapy increase healthcare costs and require more frequent patient visits [6]. Addressing these conditions is essential to maintaining effective cancer treatment protocols.

### 4.1. Chemotherapy-Induced Neutropenia (CIN) and GCSF Use

CIN is characterized by a reduction in absolute neutrophil count (ANC) below 1000 cells/μL following chemotherapy, with severe neutropenia (grade 3) defined as ANC between 500 and 1000 cells/μL, and profound neutropenia (grade 4) as counts below 500 cells/μL [24,25,26,27,28,29]. The highest risk of CIN occurs during the first chemotherapy cycle, with over 50% of cases reported in this phase, particularly febrile neutropenia [1,30,31,32,33]. Severe CIN is closely linked to increased risks of infection, ICU admissions, and mortality [34].

GCSFs, first approved by the FDA in 1991, are glycoproteins that stimulate neutrophil production and extend their survival by activating pathways such as PI3K/AKT, JAK/STAT, and MAP kinase in neutrophil progenitors [35,36,37,38,39]. Available GCSF products for CIN management include Filgrastim, Filgrastim-sndz (a biosimilar), Tbo-filgrastim, and Pegfilgrastim [40,41,42]. Filgrastim and its biosimilars are widely used for their cost-effectiveness and dosing flexibility, while Pegfilgrastim offers the advantage of a longer half-life, reducing the risk of hospitalization for neutropenia [43,44,45,46,47,48].

While GCSFs are effective, the most common side effect reported is bone pain, which can be managed with anti-inflammatory medications. Less common side effects include transient leukocytosis, thrombocytopenia, splenic rupture, and pneumonitis [49,50]. In leukemia patients, particularly in pediatric and secondary AML cases, there has been concern over an increased risk of relapse with GCSF use [51,52]. However, conflicting data exist, and caution is advised in these cases [53,54].

For prophylactic use, ASCO and NCCN recommend GCSF when the risk of febrile neutropenia is 20% or greater [41,55]. This risk is determined by factors such as age over 65, comorbidities (e.g., HIV, immunosuppressive conditions), renal or liver impairment, performance status, and chemotherapy myelotoxicity [41,55]. Algorithms, like the one developed by Bozcuk et al., predict febrile neutropenia risk with high sensitivity and help stratify patients into risk categories [56,57]. Despite these recommendations, some studies question the impact of primary prophylactic GCSF on reducing severe CIN or infection-related mortality [34,58].

For therapeutic use, GCSF is typically administered 24–72 h after the last chemotherapy dose when ANC falls below 500 cells/μL, with or without fever, and continued until ANC stabilizes at ≥1000 cells/μL for at least two consecutive days [59,60]. GCSF has been shown to reduce the duration of grade 4 neutropenia, antibiotic use, and hospital stays [61].

### 4.2. Chemotherapy-Induced Anemia (CIA) and Use of ESAs

CIA is characterized by a decrease in hemoglobin (Hgb), red blood cell count, or packed red cell volume [62]. Anemia affects 30–90% of cancer patients and is classified into four grades based on severity [62]. CIA grade 2 or higher, defined as Hgb < 10.0 g/dL, typically requires treatment due to risks such as fatigue, decreased functional capacity, and complications [62,63,64]. Management includes red blood cell transfusions for immediate correction, though this is short-lived and carries risks. Transfusions remain necessary when Hgb falls below 7–8 g/dL [62]. Iron supplementation is often used alongside ESAs to enhance treatment efficacy [62].

ESAs were approved for CIA in 1993 (epoetin alfa) and 2002 (darbepoetin alfa) [65]. These agents mimic the action of EPO, a cytokine produced by the kidneys in response to hypoxia, by binding to EPO receptors on erythroid progenitors in the bone marrow. This activates key signaling pathways like JAK2/STAT5, PI3K/AKT, and MAPK, stimulating the proliferation and maturation of red blood cells [66,67].

According to NCCN guidelines, ESAs are recommended for patients receiving palliative chemotherapy to reduce transfusion requirements and alleviate anemia symptoms [62]. Their use has been shown to improve hemoglobin levels and quality of life in patients with CIA undergoing chemotherapy with non-curative intent [62]. A study by Lai et al. reported improved short-term survival in this patient population with ESA use [68]. However, ESAs are not recommended for patients receiving curative chemotherapy due to the increased risks of thrombosis, tumor progression, and mortality [69,70]. Studies have shown a significant correlation between ESA use and venous thromboembolism (VTE), particularly when targeting hemoglobin levels above 12 g/dL [65,71]. A meta-analysis by Bohlius et al. found that ESA use was associated with a higher mortality risk, with a combined hazard ratio of 1.17 (95% CI 1.06–1.30) [72].

The 2019 ASCO and ASH guidelines recommend using the lowest effective dose of ESA, in combination with iron supplementation, for patients undergoing non-curative chemotherapy with Hgb levels below 10 g/dL [69]. This approach minimizes transfusion needs while reducing the risk of side effects [69]. The FDA also mandates that ESA use requires informed patient consent under the Risk Evaluation and Mitigation Strategy (REMS) program [62]. In certain cases, particularly in patients with chronic kidney disease (CKD) or myelodysplastic syndromes (MDS), reducing the ESA dose can improve safety without compromising effectiveness [62,69,73]. Additionally, NCCN guidelines suggest reducing the ESA dose once Hgb levels increase by 1 g/dL over two weeks or reach a threshold sufficient to avoid transfusions [62].

### 4.3. Chemotherapy-Induced Thrombocytopenia (CIT) and Use of TPO-RAs

CIT is defined as a reduction in platelet count to less than 100 × 10^9^/L due to the myelosuppressive effects of chemotherapy [74,75]. The overall incidence of CIT among patients treated with chemotherapy ranges between 9.7% and 28% [6,74]. However, certain cancer types and chemotherapy regimens were associated with significantly higher incidence of CIT, as illustrated in Table 2. CIT can manifest from mild, asymptomatic thrombocytopenia to severe, life-threatening bleeding, especially when platelet counts fall below 25–30 × 10^9^/L [6,74,76,77]. Even with platelet counts above 30 × 10^9^/L, complications can occur, especially in solid tumor patients with organ invasion [6,74,76,77]. CIT often requires chemotherapy dose adjustments or reductions, leading to lower relative dose intensity and inferior oncologic outcomes [6,11,76,77,78,79,80,81,82]. The impact of CIT extends beyond physical complications, affecting patients’ quality of life and performance status, as they may experience critical bleeding, frequent hospitalizations, and transfusions [76,77]. This, coupled with the associated poor oncological outcomes and reduced overall survival, causes significant financial burdens. One study estimated that CIT patients’ average inpatient care cost was approximately USD 36,448 [6].

Management of CIT typically involves platelet transfusions, chemotherapy dose adjustments, and treating any underlying factors. In some cases, TPO-RAs are used [11,85,86,87]. Oprelvekin, a recombinant interleukin 11, was previously used but was withdrawn due to cardiovascular side effects [88,89].

Although there are no FDA-approved TPO-RAs for CIT, TPO-RAs like romiplostim and eltrombopag have shown efficacy based on their success in treating immune thrombocytopenia (ITP) [77]. The NCCN guidelines support the use of TPO-RAs for off-label CIT management [11,88,90]. Romiplostim, a peptibody administered weekly by subcutaneous injection, mimics thrombopoietin by binding to the thrombopoietin receptor (TPO-R) on megakaryocyte precursors, stimulating platelet production [91,92,93]. In a phase II trial, 93% of patients treated with romiplostim achieved platelet counts averaging 141,000/µL after two weeks [94]. Eltrombopag is an oral, non-peptide small molecule that binds to the transmembrane domain of TPO-R, initiating the signaling cascade responsible for megakaryocyte proliferation and differentiation [91,92]. It is FDA-approved for ITP, hepatitis C-associated thrombocytopenia, and severe aplastic anemia [95,96]. These first-generation TPO-RAs were approved by the FDA in 2008 for chronic ITP [97]. Other newer TPO-RAs include avatrombopag and lusutrombopag. Avatrombopag is an orally administered TPO-RA approved for ITP and periprocedural thrombocytopenia in chronic liver disease patients [77,98]. Studies have shown promising results, with platelet recovery in 11 to 50 days, with a median of 27.5 days [99]. Another study showed recovery to ≥75 × 10^9^/L within 9.4 days and ≥100 × 10^9^/L in 10.2 days [100]. Lusutrombopag, another oral TPO-RA, is FDA-approved for periprocedural thrombocytopenia in chronic liver disease patients [77,101].

Recombinant human thrombopoietin (rhTPO), a glycosylated polypeptide that mimics endogenous TPO, works by stimulating the differentiation of bone marrow stem cells into megakaryocyte progenitors [102,103,104]. It is administered subcutaneously once weekly [105,106,107,108,109]. Although rhTPO is not FDA-approved for CIT, studies have shown that administering it five days before chemotherapy significantly reduces the severity of early thrombocytopenia [110,111].

TPO-RAs have been effective in managing CIT, but concerns remain about side effects, particularly the risk of thrombosis. This risk is especially relevant in CIT patients, who are already in a hypercoagulable state due to cancer. However, studies have shown that long-term use of TPO-RAs (over one year) does not significantly increase thromboembolic events [112,113,114,115,116]. Another concern is the risk of myelofibrosis (MF). While some studies suggest that TPO-RAs may induce reticulin formation in the bone marrow, discontinuing treatment generally reverses this effect [117,118,119]. The risk of MF remains low and is more likely after prolonged use [117,118,119]. When selecting a TPO-RA, factors such as availability, safety profile, cost, and patient preferences should be considered. For instance, eltrombopag may cause hepatotoxicity and should be avoided in patients with liver conditions [95]. Additionally, patient preference for oral versus injectable formulations and dosing frequency should be considered.

## 5. Cancer-Related Risk with the Usage of Growth Factors in CIC

In addition to the known adverse events that could increase in incidence in patients with underlying malignancies, such as thrombosis, as discussed earlier, concern for malignant progression in patients receiving CIC has been reported. The use of GCSF in solid malignancies, such as lung and breast cancers, is likely associated with an increased risk of therapy-related hematological malignancies, particularly acute myeloid leukemia and myelodysplastic syndrome [120,121,122]. In a meta-analysis of 68 randomized trials across various malignancies, the authors found the use of GCSF to be associated with a higher incidence of hematological malignancies (relative risk 1.85, 95% confidence interval 1.12 to 2.88). However, there was also a reduction in mortality, especially in patients receiving dose-dense chemotherapy regimens, likely due to reduction in myelosuppression [120]. A French nationwide population-based report on breast cancer indicated that GCSF use increased the risk of acute myeloid leukemia, myelodysplastic syndrome, and acute lymphocytic leukemia [122]. Although the risk of therapy-related hematological malignancy with GCSF plus chemotherapy is present, the absolute magnitude of the risk is small, and it is likely outweighed by the benefit of using GCSF in this setting. Nonetheless, the prescribing information has been modified in a supplemental approval to indicate the risk of myelodysplastic syndrome and acute myeloid leukemia following chemotherapy or radiation, particularly in patients treated for lung cancers, with recommendations to monitor for signs and symptoms of hematological neoplasms [123,124]. The increased risk of secondary AML/MDS in patients with solid malignancies may partially result from the intensified chemotherapy, which necessitate increased GCSF support. However, distinguishing whether this elevated risk arises from the direct effects of GCSF or the underlying myelosuppressive chemotherapy remains challenging [125].

Limited data exist regarding the risk of malignant progression with TPO-RA, but the available evidence suggests the absence of TPO receptor expression in most solid tumors. However, we recommended evaluating for TPO receptor (MPL), particularly in hepatocellular carcinoma, when available, to confirm the absence of MPL mRNA or protein expression before commencing TPO-RA usage [126,127].

As for ESAs, as discussed earlier, their use has been discouraged in patients receiving chemotherapy with curative intent due to the lack of survival benefits and a possible increase in mortality. Different tumor types and cell lines have demonstrated the presence of EPOR mRNA transcript in cancer stem cells, with protein expression confirmed in most tumor cell lines [128,129,130,131]. Despite many tumors expressing EPOR, the effect of EPO on these cells remains debated. Some studies report a proliferative response of cancer cells after EPO exposure, while others do not, though chemotherapy resistance may increase [132,133]. A randomized, placebo-controlled trial examining epoetin in head and neck cancer patients with anemia undergoing radiotherapy found that while epoetin corrected the anemia, it increased tumor progression and reduced progression-free survival [134]. Another placebo-controlled trial by the Danish Head and Neck Cancer using darbepoetin alfa was terminated early due to significantly poorer tumor control and survival in the ESA-treated cohort [135]. Similarly, the multicenter, randomized, double-blinded, placebo-controlled Breast Cancer Erythropoietin Trial (BEST) of women with metastatic breast cancer receiving frontline chemotherapy was terminated due to increased tumor progression and vascular events [136]. In conclusion, numerous reports indicate that erythropoietin may promote tumor growth and progression through the growth-promoting and anti-apoptotic effects. As a result, its use is generally not recommended in patients with CIA treated with a curative intent.

## 6. Predictors of Response to Growth Factors in CIC

In the case of CIN and the use of GCSF, studies have shown that patients with higher leukocyte peaks after GCSF administration tend to experience a lower incidence of febrile neutropenia [137].

For CIA and ESAs, several markers have been identified to predict response, most of which have been inspired by their role in MDS. These include the baseline EPO level, early changes in soluble transferrin receptors, the Revised Erythroid Dysplasia (RED) score, and the hepcidin/ferritin ratio [138,139]. A baseline serum EPO level exceeding 500 IU/L may suggest avoiding ESA use, as the response is likely to be suboptimal [69]. The RED score is derived from flow cytometry and evaluates the degree of dyserythropoiesis; a score greater than 4 indicates a poorer response to ESAs [138]. While these markers were primarily studied in MDS patients, they may also be valuable in CIA management.

As for CIT and TPO-RAs, lower baseline serum TPO level have been associated with a better response to romiplostim [140].

## 7. Future Insights and Ongoing Clinical Trials

G-CSF is already approved for the treatment of CIN and is widely used for both the prophylaxis and treatment of febrile neutropenia. A potential direction for future clinical trials would be to explore its use for the prevention of CIN, which could further enhance patient outcomes.

In the context of CIA and ESAs, there is an ongoing clinical trial (NCT05768997) evaluating the use of high-dose intravenous iron in combination with ESA for patients receiving chemotherapy with palliative intent. Another area worth exploring in future studies is whether reduced ESA doses and hemoglobin targets (e.g., 8 g/dL) can be utilized in CIA patients undergoing chemotherapy with curative intent, aiming to minimize side effects without compromising treatment efficacy.

It is worth noting that other treatments are being explored in CIA, including Roxadustat, a hypoxia-inducible factor prolyl hydroxylase (HIF-PH), in the setting of CIA which has shown promising results [141].

For CIT and TPO-RAs, several ongoing trials are investigating their efficacy and safety for both treating and preventing CIT (Table 3). In addition, future research should focus on identifying further biomarkers that could predict which patients are more likely to respond to growth factors in the setting of CIC, particularly in cases of CIA and CIT.

One area of ongoing research involves the presence of genetic variations that may increase the risk of developing CIC. Bjorn et al. studied the genetic variations in patients with non-small cell lung cancer treated with gemcitabine/carboplatin and their association with CIT. Based on these factors, a weighted genetic risk score has been developed to predict CIT risk (Table 1) [19].

## 8. Conclusions

CIC remains a significant complication in cancer treatment, affecting both patient quality of life and overall treatment efficacy. The strategic use of growth factors, such as GCSF, ESAs, and TPO-RAs, offers a promising avenue for mitigating these effects. However, due to the complexity of CIC, growth factors must be used with caution. Tailoring the administration of these agents to balance their therapeutic efficacy with potential risks, such as thrombosis or relapse, is essential to achieving optimal outcomes. Ongoing research and clinical trials will continue to refine their application, ensuring that these treatments offer durable and safe solutions for cancer patients experiencing CIC.

## Figures and Tables

**Table 1:** Risk factors for significant or persistent chemotherapy-induced cytopenia.

Risk Factors	Details
Disease-related factors	Hematological malignancies [1]. Certain solid tumors; lung and bladder cancers [11,12]. Pre-existing cytopenia before starting treatment [1]. Massive splenomegaly [1]. Bone marrow involvement by disease or extensive fibrosis [13].
Patient-related factors	Age > 65 years [1,11,13,14]. Baseline cytopenia [13]. Renal or liver impairment [1,11,13,14,15] *. Medications that may interact with chemotherapy [1,15,16]. Malnutrition and hypoalbuminemia [1,14,17,18]. Concurrent or recent infections which can lead to myelosuppression [12]. Genetic factors ** [19].
Treatment-related factors	High doses and combination therapies [1,20,21]. Combining chemotherapy with radiation [1]. Previous use of chemotherapy or radiotherapy [11]. Type of cytotoxic agent *** [1,18,21,22,23].

* Renal and liver impairment can disrupt the clearance and metabolism of chemotherapy, which results in prolonged presence of them in the body, which might increase the toxicity from it [1,14]. ** Several single-nucleotide variants (SNVs) genes were found to be associated with CIT in patients with non-small cell lung cancer treated with gemcitabine/carboplatin in a study by Bjorn et al. These genetic factors include SNVs in JMJD1C and DOCK8, SERPINA5, SERPINC1, and CAPZA2 genes [19]. *** Alkylating agents, antimetabolic agents, antineoplastic antibiotics, plant-derived alkaloids, and platinating agents [18].

**Table 2:** Types of cancers and chemotherapy regimens used that are most commonly associated with CIT.

Cancer Types/ Chemotherapy	Details		Percentage of Patients with CIT (by Chemotherapy Regimen)	Intervention Used for CIT
Cancer types	Diffuse Large B-cell Lymphoma (DLBCL) with the following regimens [83]	DHAP	92.3%	Platelet transfusion
		ICE	89.7%	
		GDP	89.7%	
	Bladder cancer; especially with the use of cisplatin/gemcitabine [12]		57%	Platelet transfusion, chemotherapy dose reduction, delay or cessation
	Lung cancer; with the following treatments [12]	Carboplatin/gemcitabine	29%	
		Cisplatin/etoposide	18%	
	Nasopharyngeal carcinoma with gemcitabine and platinum compounds [84]		5.21%	Change, reduction in or cessation of chemotherapy
Chemotherapy regimens	Platinum-based regimens [75]	Carboplatin monotherapy	81.8%	Not detailed
		Combination therapies involving carboplatin	58.2%	
	Oxaliplatin combination regimens [75]		28.6%	
	Gemcitabine-based regimens [6]		13.5%	Platelet transfusion, TPO-RA (eltrombopag, used in a small number), glucocorticoids, and immunoglobulin

CIT: Chemotherapy-induced thrombocytopenia; DHAP: dexamethasone, high-dose cytarabine, and cisplatin; ICE: Ifosfamide, carboplatin, and etoposide; GDP: gemcitabine, dexamethasone, and cisplatin; TPO-RA: Thrombopoietin receptor agonist.

**Table 3:** Summary of some of the existing and ongoing trials on the use of GFs in CIC.

Growth Factor	Source/Trial	Study Details	Outcomes
GSCF	SPROG Trial, 2015 [142]	Assessed secondary prophylaxis with GCSF in breast cancer patients	GCSF significantly improved the achieved of planned RDI and reduced post-randomization neutropenic events.
	Huang et al., 2018 [60]	Compared the efficacy and safety of PEG-rhG-CSF with daily rhG-CSF in breast cancer patients	Single injection of PEG-rhG-CSF had similar efficacy to daily rhG-CSF in the reduction of the occurrence and duration of profound neutropenia (grade 4).
	PANTHER phase III Trial, 2020 [143]	Explored FN, chemotherapy dose reduction and delays with GCSF administration in two groups of breast cancer patients	GCSF use significantly reduced neutropenic events and chemotherapy delays, which allowed increased relative dose intensity.
	Najafi et al., 2021 [144]	Compared efficacy and side effects of pegfilgrastim with filgrastim in breast cancer patients	Pegfilgrastim is non-inferior to filgrastim, with less toxicity.
	ADVANCE Trial, 2022 (NCT02643420)	Compared the efficacy and safety of SPI-2012 (eflapegrastim) vs. pegfilgrastim in breast cancer patients treated with TC to assess the duration of severe neutropenia in cycle 1	Eflapegrastim was non-inferior to pegfilgrastim.
ESA	Henke et al., 2003 [134]	Compared epoetin beta versus placebo in head and neck cancer patients with anemia who received curative radiotherapy +/− resection	Epoetin corrected anemia but increased tumor progression, and reduced PFS.
	BEST Trial, 2003 [136]	Investigated the effect of epoetin alfa to prevent anemia in patients with metastatic breast cancer	Terminated early due to higher mortality in the ESA group.
	DAHANCA 10 Trial, 2018 [135]	Patients with squamous cell carcinoma of the head and neck randomized to receive darbepoetin alfa vs. no ESA	Correction of Hgb levels with ESA during radiotherapy resulted in a significantly poorer tumor control and survival.
TPO-RA	Vadhan-Raj et al., 2003 [103]	The use of rhTPO before and/or after cycle 2 of chemotherapy in sarcoma patients	rhTPO given early (day −5) significantly reduced the severity of CIT.
	Soff et al., 2019 [94]	Phase II trial to studied romiplostim in patients with solid tumors and CIT	The majority of the romiplostim-treated group achieved platelet correction and resumed chemotherapy without recurrent CIT.
	Frey et al., 2019 [145]	Studied combining eltrombopag with induction chemotherapy in AML, except for M3 and M7 subtypes	Higher rates of side effects and mortality were observed in patients who received eltrombopag.
	Shin et al., 2023 [146]	Murine study, innvestigated a novel TPOr agonist in a mouse model with CIT	2R13 significantly increased platelet counts and sustained higher platelet levels compared to rhTPO.
	RECITE Trial (NCT03362177)	The use of romiplostim to treat CIT in patients with GI, pancreatic, or colorectal cancers	Ongoing trial
	PROCLAIM Trial (NCT03937154)	The use of romiplostim for CIT in adults with non-small cell lung cancer, ovarian cancer, or breast cancer.	Ongoing trial
	ACT-GI Trial (NCT05772546)	The use of Avatrombopag in CIT in patients with GI malignancies	Ongoing trial (recruiting)
	NCT06099925	The use of Hetrombopag in CIT in gynecological malignancies	Ongoing trial (not yet recruiting)
	NCT06521931	The safety, tolerability, pharmacodynamic and kinetics, along with immunogenicity of the novel TPO-RA (PN20) to prevent CIT in patients with lymphoma and solid tumors	Ongoing trial (recruiting)

## Data Availability

The authors declare that data supporting this study’s findings are available within the article.
